# The role of insight into and beliefs about medicines of hypertensive patients

**Published:** 2007

**Authors:** Clarris Shiri, Sunitha C Srinivas, William T Futter, Sarah E Radloff

**Affiliations:** Pharmacy Administration and Practice, Faculty of Pharmacy, Rhodes University, Grahamstown; Pharmacy Administration and Practice, Faculty of Pharmacy, Rhodes University, Grahamstown; Pharmacy Administration and Practice, Faculty of Pharmacy, Rhodes University, Grahamstown; Department of Statistics, Rhodes University, Grahamstown

## Abstract

**Objectives:**

One aim of this study was to determine the level of knowledge and understanding of selected individuals about hypertension, as well as their beliefs and perceptions about medicines. The other purpose was to determine the medicines information provision system that these participants were exposed to.

**Methods:**

Participants filled in the Beliefs about Medicines questionnaire and one-on-one interviews were conducted. Participants gave informed consent and their health passports were examined. A focus-group discussion was held with some of the nurses at one of the local clinics.

**Results:**

Participants believed their antihypertensive therapy was necessary for them to maintain their health. However, there was also a high level of concern about the undesirable effects of the medication. Most participants did not understand what hypertension is, however, they were aware of the consequences of uncontrolled blood pressure. There was no structured patient education system at the public clinic investigated.

**Conclusion:**

A knowledge gap existed which needed to be filled. Participants’ concerns about the undesirable effects of antihypertensive therapy needed to be addressed. A structured medicines information provision system is required at the public clinic studied, to ensure that patients receive all the pertinent information about their condition, namely hypertension, and the prescribed therapy.

## Summary

Levels of non-adherence to anti-hypertensive agents are very high^1^-^4^ and it is one of the main causes of poor blood pressure control.^5^-^7^ Stroke, one of the complications of uncontrolled blood pressure, kills more people than communicable diseases such as AIDS.^8^-^11^ Considerable effort must therefore be put into reducing the risks of developing stroke as well as other complications of high blood pressure.^12^

According to the health belief model,^13^ factors such as cultural norms and knowledge about a disease modify beliefs and perceptions and can thereby influence behaviour, for example, adherence to therapy. Horne and Weinman^14^ have reported a correlation between beliefs about medicines and reported adherence. Beliefs are not static^15^ and therefore by altering factors affecting beliefs and perceptions, it is possible to influence these beliefs and in turn transform behaviour. Unless an individual believes that his/her disease or condition poses a threat to his/her life, he/she may not see the need to adhere to a prescribed therapeutic regimen.

Low levels of adherence to antihypertensive therapy are probably because high blood pressure is typically an asymptomatic condition.^16^-^19^ The benefits of therapy may therefore escape the patient’s notice simply because he/she is unaware of the alleviation of any symptoms. Unlike, for example, a skin rash, there is no constant reminder that the patient is sick if he/she discontinues therapy.

One of the greatest challenges for healthcare professionals is convincing the patient that there is a casual link between therapy and health. This is obviously much more difficult in the case of an asymptomatic condition such as hypertension. Unless the reasons for adherence are made known to the patient, he/she may discontinue the therapy. It is therefore essential that healthcare providers (HCPs) supply healthcare receivers (HCRs) with pertinent information about their condition, such as the causes, diagnosis, treatment and prognosis.^20^ Educational interventions provide opportunities to influence patients’ attitudes towards their treatment and increase adherence to therapy.^1^,^2^,^21^,^22^

It is essential to put more effort into identifying the knowledge gap regarding what patients do know and can do, in comparison with what they need to know and be able to do.^20^ Those planning the interventions should therefore take into account the participants’ level of knowledge and understanding as well as perceptions about their condition and its management.

Beliefs about medicines fall into two categories, namely the beliefs about medicines specifically prescribed for the patient, and those beliefs about the general nature of all medicines.^14^ The specific beliefs are further divided into two: (1) the individual’s perception of the necessity of his/her prescribed therapy for maintaining his/her health and preserving his/her life; (2) the respondent’s concerns about the potentially undesirable effects of his/her therapy.

The beliefs about medicines have been measured using the Beliefs about Medicines questionnaire (BMQ).^23^ This is a reliable instrument, designed by Robert Horne and his colleagues in the UK, which measures patients’ beliefs about medicines, both general and specific. A person weighs the benefits of prescribed therapy against its potential risks.^14^,^24^ An interaction between these two factors is known as the necessity−concerns differential (NCD).^23^ This is calculated as the difference between the score for perception of necessity and the concerns about the prescribed therapy. A high positive value indicates that the perception of the necessity of the therapy outweighs the concerns about the undesirable effects. Being aware of the healthcare receivers’ beliefs and levels of knowledge and understanding will increase the probability that the educational programme will be effective.

The primary aim was to determine the level of knowledge and understanding about hypertension of selected hypertensive patients. The secondary aim was to establish the level of reported adherence to therapy.

## Methods

## Study design

A baseline study was carried out to find out how much the participants knew about hypertension and its therapy. Participants were recruited via letters of invitation from a defined group, namely support staff members at Rhodes University. The people targeted were in the low-income bracket and did not have access to most sources of medical information such as books, journal articles and the Internet. A focus-group discussion was held with three nursing sisters from one of the clinics from where the majority of the participants received their chronic therapy. This was to determine if there was a structure in place for patient education.

The letters invited hypertensive volunteers to take part in a programme where they would learn more about their condition. The letters explained that the programme involved interviews and talks as well as provision of written information about hypertension and its management, both pharmacological and non-pharmacological. The project received approval from the Ethics committee at Rhodes University. Participants gave written informed consent.

Participants were accepted in the programme if they were hypertensive and on anti-hypertensive therapy. They also had to be working for the University as support staff members throughout the study. Individuals were excluded if they had previously been diagnosed as hypertensive but had stopped therapy and were not willing to resume it. Those who had stopped therapy as per doctors’ instructions were also not included.

## Measurements

The participants were asked to fill in the Beliefs about Medicines questionnaire.^23^ This questionnaire had been translated into isiXhosa, the local language, from English. A different person, who had not seen the original English version, then translated the questions back into English. This was in order to verify the authenticity of the first translation into isiXhosa.

Each participant received both the English and the isiXhosa questionnaires and had to choose which language to respond in. The responses to the BMQ were scored according to a Likert five-point scale. The possible responses were as follows: strongly agree (5), agree (4), uncertain (3), disagree (2), and strongly disagree (1).

The mean and standard deviation values of perception of necessity of medication, concerns about the prescribed therapy, and necessity−concerns differentials were determined. The statements relating to necessity of medication were denoted by ‘N’ and those for concerns by ‘C’. The statements relating to the harmful nature of medicines and overuse by HCPs were denoted by ‘H’ and ‘O’ respectively.

Also a one-on-one interview questionnaire was designed and a pilot study was done to check for and correct any flaws in this questionnaire. There were five participants involved in this part of the study. This questionnaire was used to establish the participants’ levels of understanding about hypertension, that is, the condition itself, therapy and necessary lifestyle changes. The participants were asked both open- and closed-ended questions. For the open-ended questionnaires the responses were categorised as either correct or incorrect. Their health passports* were checked to establish their prescription refill patterns and their prescribed therapy. The interviews were conducted with an interpreter present for individuals who did not fully understand English, since the principal investigator did not speak isiXhosa.

Cronbach alpha was used to test the internal consistency reliability of the BMQ.^25^,^26^ Dependent *t*-tests were used to test for significant differences between the mean values of the perception of necessity and the concerns about the prescribed therapy.

## Results

**Fig. 1.**
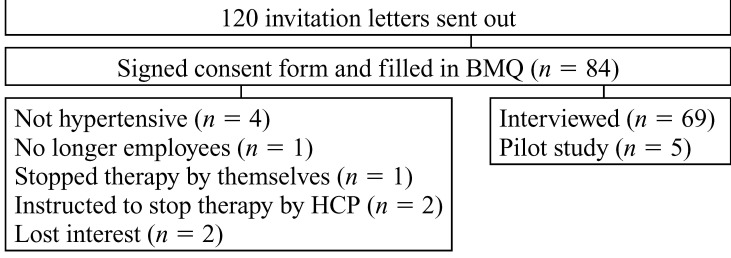
Flow of participants.

A total of 84 people responded to the invitation letters and 74 were eligible for the study. Of these, five were involved in the pilot study for the interview questionnaire and the remaining 69 participants filled in the Beliefs about Medicines questionnaire (68 filled in the section on specific beliefs and 66 filled in the section on general beliefs)^23^ and were interviewed. The other 10 did not meet the eligibility criteria (Fig. 1). Forty-seven (68.6%) of the participants were female and their ages ranged from late 20s to early 60s (Table 1).

**Table 1:** Frequency Of Characteristics Of Study Group

*Characteristics*	*Number of participants (n = 69)*	*Percentage*
Gender:		
Female	47	68
Male	22	32
Age (years):		
25−40	7	10
41−60	60	87
> 60	2	3
Race:		
Black	60	87
White	1	1
Coloured	8	12
Home language:		
English	3	4%
isiXhosa	59	86
Afrikaans	7	10
Highest qualification:		
Grades 1−4	4	6
Grades 5−7	16	23
Grades 8−12	48	70
> Grade 12	1	1
Co-existing chronic conditions:		
Diabetes	9	13
Asthma	4	6
Epilepsy	2	3
Arthritis	8	12
Gout	8	12
Gastric ulcers	5	7
Fibromyalgia	1	1
None	39	57

The internal consistency reliability (Cronbach’s alpha) for the sum scale of 10 items on the patients’ perceptions and beliefs about their medication was 0.81 and for the sum scale of eight items on the patients’ views about medicine in general was 0.75. The responses to these BMQ items, therefore, may be considered as reliable and the computed means scores for BMQ specific and general scales are shown in Table 2, along with the percentage of the sample attaining scores above the scale mid-point.

**Table 2:** Means And Standard Deviations Of Scores On BMQ Scales

*Variable*	*Number of items in scale*	*Mean*	*SD*	*Percentage of patients with scores above scale midpoint*
Specific − necessity	5	21.2	3.3	95.6
Specific − concerns	5	17.3	4.1	58.8
General − harm	4	11.3	2.9	27.2
General − overuse	4	14.2	3.3	65.2

The majority of the people agreed that their health, now (88.2%) and in the future (79.4%), depended on their medications and that life would be impossible without them (83.8%). About 72% were worried about becoming too dependent on their medications. The mean and standard deviation scores for necessity and concerns were 21.2 ± 3.3 and 17.3 ± 4.1, respectively. The mean necessity−concerns differential^23^ and standard deviation were 3.9 ± 3.9. There was a significant positive difference in mean scores between the perception of necessity and the concerns about the prescribed therapy (dependent *t*-test: *t* = 5 8.3, df = 67, *p* < 0.0001), which indicated that the perception of the necessity of the therapy significantly outweighed the concerns about the undesirable side effects. Most of the patients (95.6%) attained necessity scores above the scale midpoint and 58.8% of them attained concerns scores above the scale midpoint (Table 2).

The mean and standard deviation scores for the harmful nature of medicines and overuse by HCPs were 11.3 ± 2.9 and 14.2 ± 3.3, respectively. Twenty-six (39.4%) and 50 (75.8%) of the participants who filled in the BMQ felt that doctors used too many, and put too much trust in medicines, respectively. Some of the patients (27.2%) attained harmful scores above the scale midpoint and 65.2% of them attained overuse scores above the scale midpoint (Table 2). Thirty-eight (57.6%) of them believed that if consultation times were longer, fewer medications would be prescribed. Forty-five of the participants (68.2%) thought that most medicines were addictive. Eight (12.1%) and 12 (18.2%) perceived all medicines as being poisonous or causing more harm than good, respectively.

The frequencies of responses to some of the questions asked during the one-on-one interviews are shown in Tables 3 and 4. The majority of the participants (95.6%) could not define hypertension. Many of them knew it was associated with certain lifestyle factors such as diet, exercise and stress. Stroke was mentioned by the majority as a consequence of uncontrolled hypertension. Not many were aware that the prognosis also includes heart and kidney failure, dementia and even death.

**Table 3:** Frequency Of Responses To Interview Questions Relating To Self-Reported Adherence (*n* = 69)

*Questions*	*Correct*	*Yes*	*Incorrect*	*No*
How do you take your medication for high blood pressure?	60		9	
What do you do when you miss a dose?	64		5	
Do you take your medication all the time even when you feel well?		59		10
Do you ever forget to take your medication?		32		37
Besides forgetting, are there any other reasons why you might not take your medication as you were told to?		9		60

**Table 4:** Frequency Of Responses To Intervi Ew Questio Ns Relating To Knowledge About Hypertension (*n* = 69)

*Questions*	*Correct*	*Yes*	*Incorrect*	*No*	*Don’t know*
What is hypertension?	3		2		64
What is the desirable blood pressure?	8		5		56
What will happen if your blood pressure is not controlled?	Stroke (40)				
	Heart attack (4)				
	Death (4)				
	Other cardiovascular problems (1)				
What are the names of your medications for high blood pressure?	16				53
How long are you going to be taking medication for high blood pressure?	37		3		29
Do you think that if you feel fine then your blood pressure is also fine?		23		46	
Do you know that there are some foods you cannot eat or should eat in small amounts because of your high blood pressure?		52		17	
Do you think that your medication alone is enough to control your high blood pressure without you having to change your lifestyle?		24		44	1
Do you think there is a cure for high blood pressure?		8		45	16
Do you know that there are some medications you cannot take because you have high blood pressure, for example some ’flu medicines?		23		46	

For nine (13.0%) of the participants there were discrepancies between the dose of medication stated on the packaging and the dose reported by the individuals. After checking the health passports, it was realised that the dosing on the packaging was in accordance with that in the health passport. The mistake was therefore on the HCR’s part. There were 64 (92.8%) participants who reported that if they missed a day’s dose for one reason or the other, they did not compensate for it on the next day but just took the normal dose as usual.

Out of the whole group, 17 (24.6%) participants stated that they were not aware of the need to alter their diet after they had been diagnosed with high blood pressure. They reported that they had not been told anything when they were diagnosed. Half of them said that their friends and colleagues had told them that there were certain foods that hypertensive individuals are not supposed to eat.

Besides forgetting (46.4%), other reasons listed for failure to take medication as directed included the patient feeling it was not necessary or it was not working, getting alternative therapy from traditional healers, and one said he had not been told to come back after getting the initial supply and as he was not aware that the therapy was meant to be for life, he had not returned to get more. He probably assumed that he had a short-lived illness with short-term therapy. The reason he was included in this study was that he responded to the invitation letters because the nurses had in the past mentioned that he had had high blood pressure. The principal investigator only learnt during the course of the interview that this participant was not taking any medication for hypertension.

More than half of the participants (53.6%) were aware that their therapy was for life; however 29 (42.0%) did not know how long they were going to be on anti-hypertensive medication. Only 23 (33.3%) seemed to be aware of potential interactions between other drugs and their hypertension. From the health passports, it was determined that a large number (72%) of the participants had been collecting their prescription refills regularly for the six months prior to the interview. However some of them (54%) did not always collect their refills on time.

From the focus-group discussion, it was established that the clinic did not have adequate structures in place for patient education and counselling, due to lack of resources such as materials, time and manpower. For example, there was no checklist for the points which the nurses had to address when counselling a patient diagnosed with hypertension for the first time.

* Health passport: a book where all details about the patient’s visits to public healthcare centres are recorded. The patient keeps this book. The private sector does not use this system.

## Discussion

The dependent *t*-test results showed that the perception among these participants of the necessity of antihypertensive medication outweighed their concerns about the undesirable effects of the medication. However, the mean score for concerns was relatively high, indicating that a number of concerns did exist and needed to be addressed. The Cronbach alpha values showed that the participants’ responses could be considered as reliable.^25^

If patients do not understand the true nature of hypertension they can believe that at some point they will be cured and be able to stop taking their antihypertensive medication. It is possible that HCPs can make incorrect assumptions about the amount and type of knowledge possessed by HCRs. For example, in this study, 17 participants did not know that they were supposed to alter their diet after diagnosis with hypertension. One participant had not been told that he was going to be on antihypertensive medication for life. The above are examples of poor adherence due to lack of pertinent information from HCPs.^22^ This can lead to therapeutic failure and even complications of the condition which could have been avoided.

Based on the findings from the focus-group discussion, one can deduce that the nurses can, understandably, forget to address certain topics when providing the HCRs with information concerning their condition. There was no written information at this clinic that patients could take home. The latter therefore relied on what the HCPs told them and what they heard from friends and neighbours. Information from these other sources could have been false or incomplete. Another problem is illiteracy; a lot of written information is in English and sometimes without enough visual aids to explain what is written.

One of the common discrepancies found was that patients were taking half a tablet daily of the drug hydrochlorothiazide, instead of the recommended one tablet daily. This was probably because some of their colleagues had been told to take half a tablet daily. It did not come as a surprise that some people (46.4%) reported forgetting to take their medication on some days. For most, forgetfulness was the only reason given for failing to take their medication.

More than half of the participants (63.8%) reported being aware that their medication alone was not enough and that they also needed to alter their lifestyle in order to keep their blood pressure under control. However, not all of them possessed the necessary knowledge to change their lifestyle appropriately.

The limitation of this study was that the findings reported here are based on this one group of participants at Rhodes University. They can therefore not be extrapolated to all patients served by the public healthcare system in South Africa. However, other researchers and HCPs also face similar challenges to what has been described. These include patients’ lack of knowledge about hypertension and its therapy, poor blood pressure control, poor patient adherence to therapy, and concerns about undesirable side effects of medicines. Another major challenge is inadequate patient education systems, particularly in the under-resourced countries.^27^-^34^

## Conclusion

Although a considerable number of the participants in this study were knowledgeable about some aspects of hypertension, there was still a gap to be filled and some misconceptions that needed to be cleared up. This project to introduce a medicines information provision system would therefore be of real benefit to them.

The topics that needed to be addressed during this project included the implications of having hypertension, and the role of medication and lifestyle modification. Those who sometimes forgot to take their medication could benefit from suggestions on simple reminders, for example storing their medication next to something they use every day, such as their coffee mug. It is also essential that these participants become aware that individuals can receive different doses or even different medications for the same condition.

Emphasis can also be placed on the fact that there is no cure for high blood pressure and hypertensive individuals have to take their medication indefinitely in order to keep their blood pressure under control. Another point which will be stressed during educational interventions is that it is important to take medication as directed by the HCPs. Although the majority of the participants in this study were aware that they should not double their doses to compensate for missed doses, this point cannot be overemphasised. This project provided an opportunity for the participants’ concerns about their condition and therapy to be addressed.

The problem of HCRs lacking sufficient information about their conditions and illnesses is not unique to this particular group. The whole of South Africa, if not the world, would do well to implement the strategies suggested above. This will play a huge role in decreasing poor adherence levels and saving healthcare resources.
